# Galactosyltransferases from *Arabidopsis thaliana* in the biosynthesis of type II arabinogalactan: molecular interaction enhances enzyme activity

**DOI:** 10.1186/1471-2229-14-90

**Published:** 2014-04-03

**Authors:** Adiphol Dilokpimol, Christian Peter Poulsen, György Vereb, Satoshi Kaneko, Alexander Schulz, Naomi Geshi

**Affiliations:** 1 Department of Plant and Environmental Sciences, Thorvaldsensvej 40, 1871 Frederiksberg, C, Denmark; 2 Department of Biophysics and Cell Biology, and MTA-DE Cell Biology and Signaling Research Group, University of Debrecen, Debrecen, Hungary; 3 Food Biotechnology Division, National Food Research Institute, 2-1-12 Kannondai, Tsukuba, Ibaraki 305-8642, Japan; 4 Present address: Fungal Physiology, CBS-KNAW, Fungal Biodiversity Center, Uppsalalaan 8, Utrecht 3584, CT, The Netherlands

**Keywords:** *Arabidopsis thaliana*, Arabinogalactan protein, Galactosyltransferase, Protein *O*-glycosylation, Golgi apparatus, Protein-protein interaction, FRET, Plant cell wall

## Abstract

**Background:**

Arabinogalactan proteins are abundant proteoglycans present on cell surfaces of plants and involved in many cellular processes, including somatic embryogenesis, cell-cell communication and cell elongation. Arabinogalactan proteins consist mainly of glycan, which is synthesized by post-translational modification of proteins in the secretory pathway. Importance of the variations in the glycan moiety of arabinogalactan proteins for their functions has been implicated, but its biosynthetic process is poorly understood.

**Results:**

We have identified a novel enzyme in the biosynthesis of the glycan moiety of arabinogalactan proteins. The *At1g08280* (AtGALT29A) from *Arabidopsis thaliana* encodes a putative glycosyltransferase (GT), which belongs to the Carbohydrate Active Enzyme family GT29. *AtGALT29A* co-expresses with other arabinogalactan GTs, *AtGALT31A* and *AtGLCAT14A.* The recombinant AtGALT29A expressed in *Nicotiana benthamiana* demonstrated a galactosyltransferase activity, transferring galactose from UDP-galactose to a mixture of various oligosaccharides derived from arabinogalactan proteins. The galactose-incorporated products were analyzed using structure-specific hydrolases indicating that the recombinant AtGALT29A possesses β-1,6-galactosyltransferase activity, elongating β-1,6-galactan side chains and forming 6-Gal branches on the β-1,3-galactan main chain of arabinogalactan proteins. The fluorescence tagged *AtGALT29A* expressed in *N. benthamiana* was localized to Golgi stacks where it interacted with AtGALT31A as indicated by Förster resonance energy transfer. Biochemically, the enzyme complex containing AtGALT31A and AtGALT29A could be co-immunoprecipitated and the isolated protein complex exhibited increased level of β-1,6-galactosyltransferase activities compared to AtGALT29A alone.

**Conclusions:**

AtGALT29A is a β-1,6-galactosyltransferase and can interact with AtGALT31A. The complex can work cooperatively to enhance the activities of adding galactose residues 6-linked to β-1,6-galactan and to β-1,3-galactan. The results provide new knowledge of the glycosylation process of arabinogalactan proteins and the functional significance of protein-protein interactions among *O*-glycosylation enzymes.

## Background

Arabinogalactan proteins (AGPs) are an abundant class of proteoglycans in plant cell walls and are implicated in the control of cell proliferation and morphogenesis [1]. Numerous studies using monoclonal antibodies have demonstrated the developmentally regulated appearance of specific glycan epitopes correlated with changes in anatomy (for examples, [2-11]). Hence subtle differences in the glycan structure of AGPs may function as markers used in coordinating developmental processes in plants. However, defined structural features of the active AGP glycans have not been identified and their molecular specificity is unknown.

The glycans of AGPs originate by post-translational modification of protein backbones catalyzed by glycosyltransferases (GTs) in the secretory pathway. The glycan structure of AGPs is heterogeneous, but commonly composed of a β-1,3-linked galactan backbone with substitution of the side chains at *O*6 positions (type II AG). The side chains are typically β-1,6-galactans, usually modified with arabinose (Ara) and less frequently with other sugars such as rhamnose (Rha), fucose (Fuc), and (4-*O*-methyl) glucuronic acid (GlcA) [12-14]. It is anticipated that more than 10 functionally distinct GTs are required to build the AGP glycans, and so far fucosyltransferases (AtFUT4, AtFUT6) [15], galactosyltransferases (AtGALT2 [16] and AtGALT31A [17]), and a glucuronosyltransferase (AtGLCAT14A) [18] have been characterized.

We have characterized an *Arabidopsis* GT encoded by *At1g08280,* which is co-expressed with *AtGALT31A*[17] and *AtGLCAT14A*[18]. This protein belongs to GT29 family in the Carbohydrate Active Enzyme database (CAZy, http://www.cazy.org) [19]. The GT29 family contains large numbers of eukaryotic and viral sialyltransferases acting on glycoproteins and/or glycolipids [20]. Several plant sequences have been placed in this family, and two of the rice sequences expressed in COS-7 cells showed sialyltransferase activity [21]. Arabidopsis has three proteins in this family (encoded by *At1g08280*, *At1g08660* and *At3g48820*). Two of them (*At1g08280* and *At3g48820*) expressed in COS-7 cells and in *Nicotiana benthamiana*, respectively, lacked sialyltransferase activity [21,22].

In this paper, we provide evidence for (i) β-1,6-galactosyltransferase (GalT) activity, encoded by *At1g08280* in the biosynthesis of type II AG structure, (ii) its interaction with AtGALT31A, and (iii) an increase of β-1,6-GalT activity by the protein complex in an *in vitro* assay.

## Results

### *At1g08280* is co-expressed with other type II arabinogalactan glycosyltransferases

The protein encoded by *At1g08280* is predicted to have a single transmembrane domain at Val5-Ile27, a typical type II membrane topology commonly found in GTs. The transcript levels are generally low in Arabidopsis throughout development, but higher during seed maturation and root development, and the gene is co-expressed with *AtGALT31A*[17] and *AtGLCAT14A*[18], which were recently identified as possessing galactosyltransferase and glucuronosyltransferase activity, respectively, involved in the glycosylation of type II AGs (GeneCAT, http://genecat.mpg.de) [23] (Additional file 1: Figure S1). Therefore, we presumed that the activity encoded by *At1g08280* may be involved in the glycosylation pathway of type II AGs, and investigated this hypothesis by biochemical assays using the protein expressed heterologously.

### Recombinant protein encoded by *At1g08280* showed galactosyltransferase activity towards type II arabinogalactan acceptors

**Figure 1:**
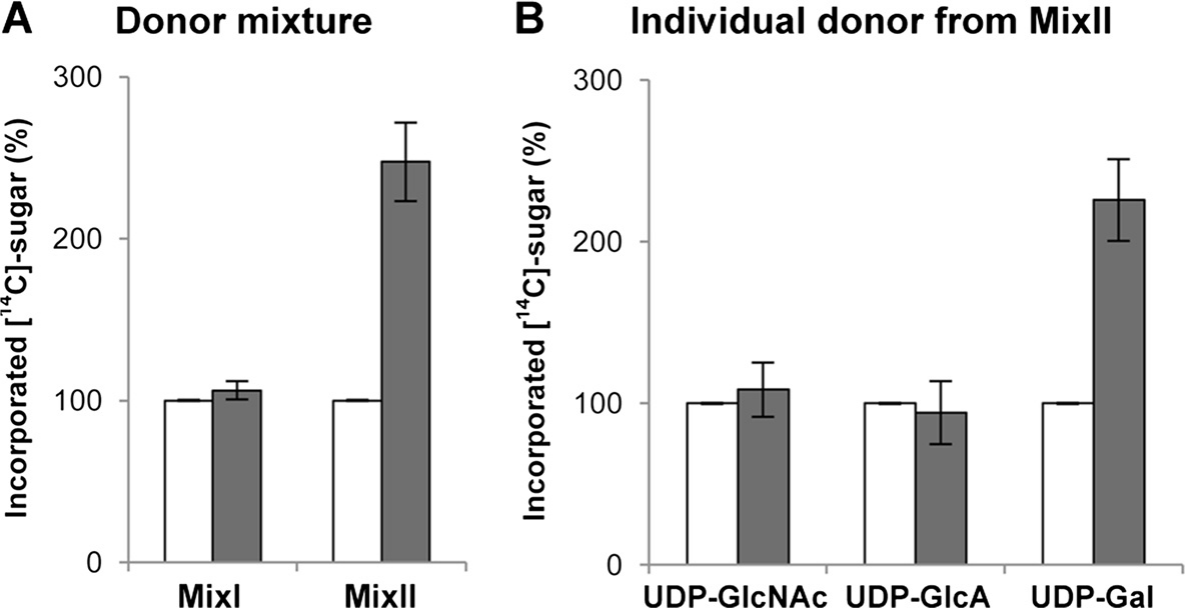
**Identification of donor substrate for recombinant AtGALT29A.** Affinity purified AtGALT29A (■) or P19 (□) was incubated with **A:** NDP-[^14^C]-sugars: UDP-[^14^C]-Xyl, UDP-[^14^C]-Glc, GDP-[^14^C]-Man and GDP-[^14^C]-Fuc (as MixI), and UDP-[^14^C]-GlcNAc, UDP-[^14^C]-GlcA and UDP-[^14^C]-Gal (as MixII); **B:** or individual NDP-[^14^C]-sugars from MixII using GAGP_8_ as acceptor substrate. Error bars showed standard deviations from n = 4. The result indicates that UDP-[^14^C]-Gal serves substrate for AtGALT29A.

For biochemical characterization, the full-length *At1g08280* construct harboring N-terminal HA tag was expressed in *N. benthamiana* and affinity purified using monoclonal anti-HA-antibody conjugated to agarose. The HA-At1g08280 collected on the bead slurry was used as the enzyme source for identification of donor substrate. We identified the donor substrate by testing 7 different NDP-[^14^C]-sugars according to the methods [17,18]. We used microsomes prepared from *N. benthamiana* after expression of a synthetic peptide composed of a consensus sequence for AG glycosylation as acceptor for the assay (GAGP_8_-GFP; [24]). This acceptor represents a mixture of various type II AG polysaccharides (for details of the structure, see [17]). When substrate mixtures were tested, we observed higher level of [^14^C]-sugar incorporation from a mixture of UDP-[^14^C]-GlcNAc, UDP-[^14^C]-GlcA and UDP-[^14^C]-Gal (Mix II in Figure 1A) than from one containing UDP-[^14^C]-Xyl, UDP-[^14^C]-Glc, GDP-[^14^C]-Man and GDP-[^14^C]-Fuc (Mix I). When testing each substrate in the Mix II separately, we found UDP-[^14^C]-Gal works as a substrate (Figure 1B). The result indicates that the enzyme possesses a GalT activity, therefore, we named the enzyme AtGALT29A (A*rabidopsis *t*haliana gal*actosyl*t*ransferase from family GT*29*).

### AtGALT29A Is localized to Golgi apparatus and interacts with AtGALT31A

**Figure 2:**
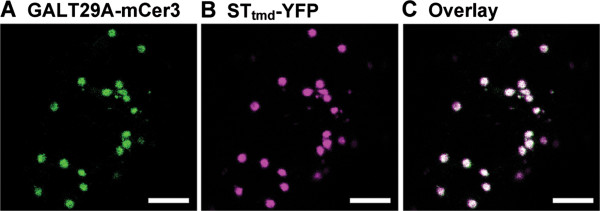
**Subcellular localization of AtGALT29A-mCer3 in *N. benthamiana *leaves. A**-**B**: Confocal images of AtGALT29A-mCer3, ST_tmd_-YFP (a Golgi marker) co-expressed transiently in *N. benthamiana* leaves. **C**: The overlay image of **(A)** and **(B)**. The result indicates co-localization of ATGALT29A-mCer3 and ST_tmd_-YFP in the Golgi apparatus. Scale bar = 5 μm.

We determined the subcellular localization of AtGALT29A by transient expression of the C-terminal monomeric CFP (mCer3) fusion protein in *N. benthamiana* (Figure 2). The overlay of AtGALT29A-mCer3 with the co-expressed Golgi marker protein, ST_tmd_-YFP [25] indicated its localization to the Golgi apparatus.

**Figure 3:**
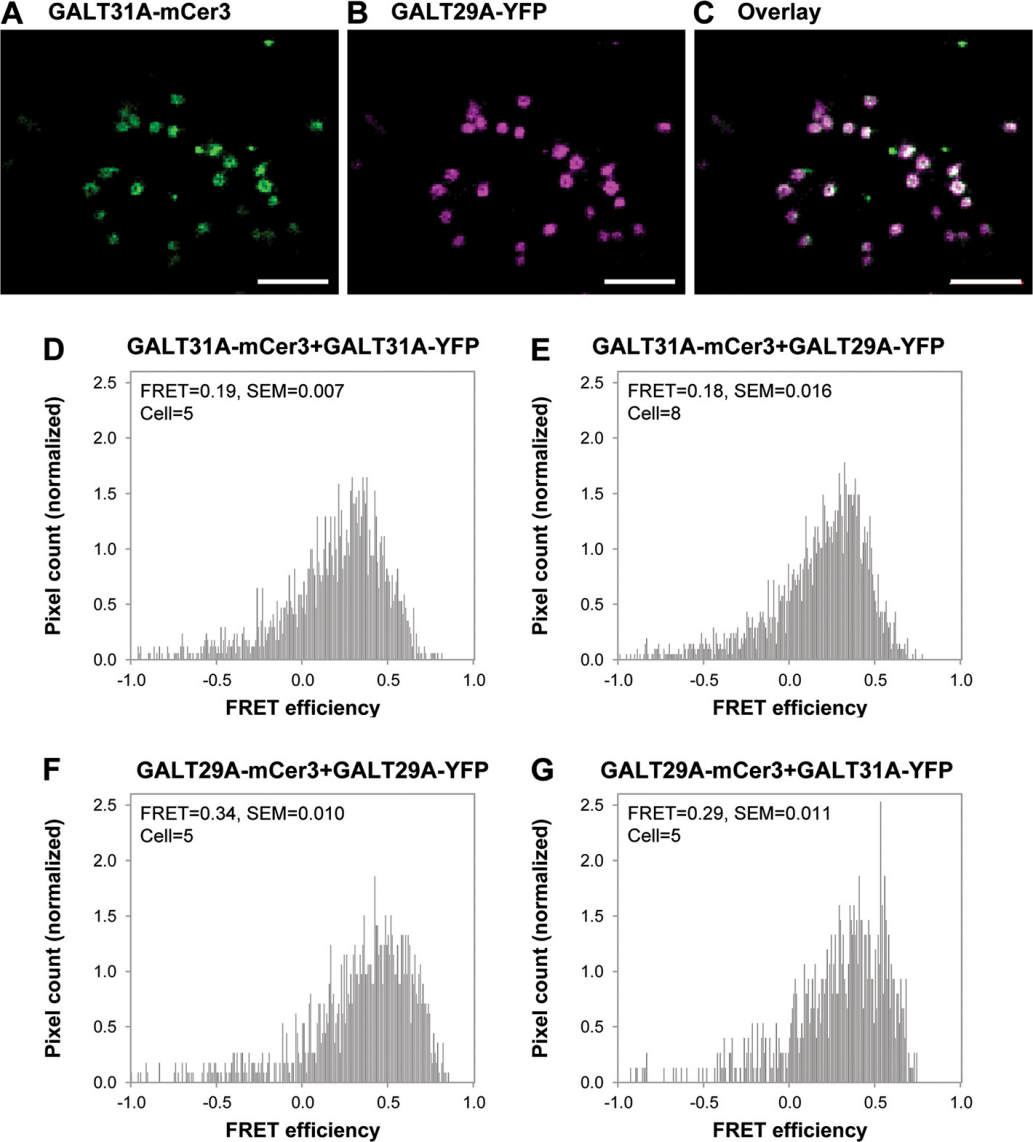
**Localization and FRET analysis for AtGALT29A and AtGALT31A. A**-**B**: Confocal images of AtGALT31A-mCer3 and AtGALT29A-YFP co-expressed in *N. benthamiana* leaves. **C**: The overlay image of **(A)** and **(B)**. AtGALT31A-mCer3 and AtGALT29A-YFP are co-localized in high frequency. **D**-**G**: Distribution histogram for pixel by pixel analysis of FRET [26]. FRET efficiency is expressed as FRET=, for example, FRET = 0.19 in **(D)** means that FRET efficiency is 19%; SEM, standard error of means; cell = number of cells analyzed. Scale bar = 5 μm.

Previously, AtGALT31A and AtGLCAT14A were also shown to be localized to the Golgi apparatus [17,18]. AtGALT29A-YFP was co-localized with AtGALT31A-mCer3 to a high degree (approximately 80%, Figure 3A-C), while AtGALT29A-mCer3 and AtGLCAT14A-YFP were only partially co-localized (approximately 52%, Additional file 2: Figure S2A-C). Next, we tested protein-protein interaction within and between AtGALT29A and AtGALT31A using the FRET acceptor photobleaching technique for these proteins tagged with either mCer3 or YFP ectopically expressed in *N. benthamiana*[26,27]. FRET from mCer3 (donor) to YFP (acceptor) happens when the two fluorescent proteins are closer than 10 nm, indicative of interaction between the tagged proteins. Bleaching of the acceptor YFP allows measuring absolute FRET efficiency in a self-controlled manner [26,27], so values above 0 definitely indicate molecular interaction between the tagged proteins. When the homodimeric combinations (AtGALT31A-mCer3 + AtGALT31A-YFP and AtGALT29A-mCer3 + AtGALT29A-YFP, respectively) were tested, FRET efficiencies of 19% and 34% were assessed, respectively (Figure 3D, 3F), indicating the formation of homodimers for both AtGALT31A and AtGALT29A. When AtGALT31A-mCer3 + AtGALT29A-YFP and AtGALT29A-mCer3 + AtGALT31A-YFP were co-expressed, FRET efficiencies of 18% and 29% were detected, respectively, indicating the formation of heterodimers between AtGALT29A and AtGALT31A (Figure 3E, 3G). Therefore we observed positive interactions for all combinations tested (Figure 3), but differences in the values of FRET efficiencies are evident, when these are calculated on a pixel-by-pixel analysis. When AtGALT29A is the donor (mCer3 tagged, Figure 3F and 3G), FRET efficiencies are overall higher (34% and 29%) compared to the combinations when AtGALT31A is the donor (19% and 18%, Figure 3D and 3E). Thus, AtGALT31A-mCer3 is either less able to dimerize than AtGALT29A-mCer3 under the experimental conditions, or is in a conformation which is less efficient as a donor. Nevertheless, when we use the same donor (either AtGALT29A-mCer3 or AtGALT31A-mCer3), and compare FRET efficiencies for homo and heterodimerization, we obtain roughly the same FRET efficiency for homo and heterodimers. For AtGALT29A-mCer3/AtGALT29A-YFP and AtGALT29A-mCer3/AtGALT31A-YFP we obtain 34% and 29%, (Figure 3F and 3G, respectively), indicating that the AtGALT29A-mCer3/AtGALT31A-YFP heterodimer is preferred to the AtGALT29A homodimer, since in spite of the possibility of homodimer formation in the AtGALT29A-mCer3/AtGALT31A-YFP system, which could decrease FRET by incorrect donor/acceptor pairing, we still have the same level of FRET efficiency as when we have only AtGALT29A. The same tendency is also observed when AtGALT31A-mCer is the donor (19% and 18%, Figure 3D and 3E, respectively).

Overall, our results indicate the formation of homodimers for both AtGALT31A and AtGALT29A as well as that of heterodimers between them when these two GTs were expressed simultaneously. The indicated interactions are unlikely to be due to an overexpression artifact since AtGALT31A and AtGLCAT14A did not interact under the same experimental set up [18]. AtGALT29A also interacted with AtGLCAT14A when the two proteins were co-localized (13% mean FRET efficiency, Additional file 2: Figure S2D). But, since AtGALT29A and AtGLCAT14A were only occasionally co-localized, occurrence of the interaction between these two proteins is considered to be of less importance than that between AtGALT29A and AtGALT31A.

### AtGALT31A is co-purified with AtGALT29A as an enzyme complex and increases the level of galactose incorporation into the type II AG acceptors

**Figure 4:**
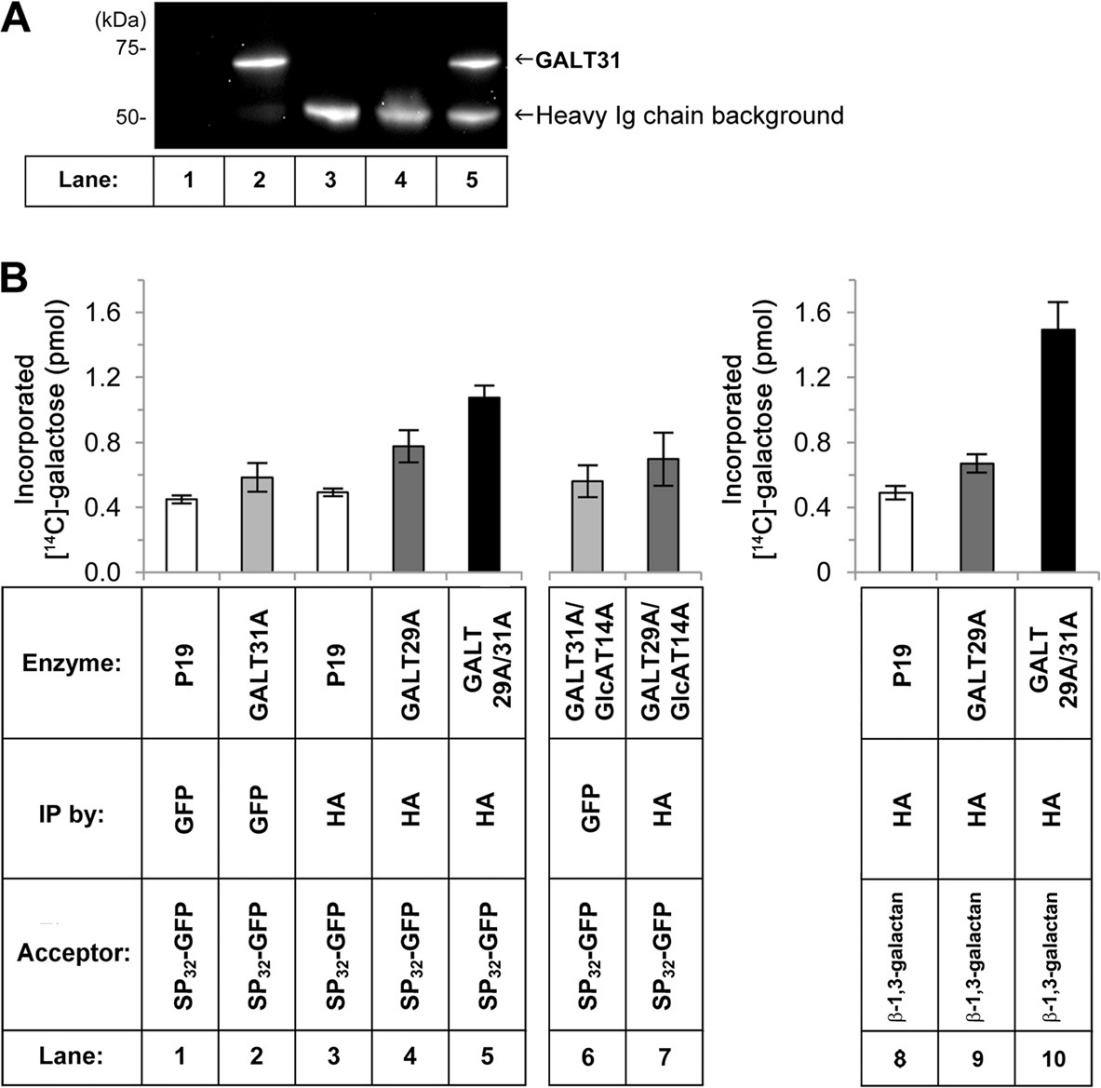
**Galactosyltransferase activity using the purified AtGALT29A/AtGALT31A complex *in vitro*.** Microsomes were prepared from *N. benthamiana* leaves after expression of P19 only, AtGALT31A-GFP, HA-AtGALT29A or co-expression of HA-AtGALT29A and AtGALT31A-GFP, and subjected to immunoprecipitation using anti-GFP- or anti-HA-antibody. The conditions are indicated in the table at the bottom of **(B)**. The immunoprecipitated samples were analyzed by the Western blot **(A)** and by the enzyme activity **(B)**. **A**: The Western blot of P19, AtGALT31A-GFP, HA-AtGALT29A and AtGALT29A/AtGALT31A immunoprecipitated using GFP antibody. The result indicates co-purification of AtGALT31A-GFP (lane 5, indicated by the arrow at ca. 70 kDa) by immunoprecipitation of HA-AtGALT29A using anti-HA-antibody-agarose. The 50 kDa band detected in the lanes 3-5 is the heavy chain of HA antibody used for the immunoprecipitation, which is recognized by the secondary antibody used in the Western blot. **B**: Galactosyltransferase activity towards SP_32_-GFP and β-1,3-galactan acceptors. Affinity purified materials from the expression of P19 only, AtGALT31A-GFP, HA-AtGALT29A, or co-expression of HA-AtGALT29A and AtGALT31A-GFP using anti-GFP- or anti-HA-antibody were tested for enzyme activity using UDP-^14^[C]-Gal as substrate and SP_32_-GFP (lanes 1-5) or β-1,3-galactan (lanes 8-10) as acceptor, (n = 4). Control samples after co-expression of AtGALT31A-GFP or HA-AtGALT29A with HA-AtGLCAT14A (lane 6 and 7) were immunoprecipitated in the same way as for other samples and tested for the enzyme activity using UDP-^14^[C]-Gal as substrate and SP_32_-GFP as acceptor (lanes 6-7), (n = 3). These combinations are not suggested to form protein complexes based on the FRET analysis. Error bars showed standard deviations.

Since FRET analysis indicated molecular interactions between AtGALT31A and AtGALT29A (Figure 3), we tried to purify the enzyme complex and investigated GalT activity when AtGALT29A is alone or in a complex with AtGALT31A. We expressed AtGALT31A as a C-terminal GFP fusion protein (AtGALT31A-GFP) and AtGALT29A as an N-terminally HA tagged protein (HA-AtGALT29A) in *N. benthamiana*, and immunoprecipitated the enzyme complex using an anti-GFP antibody (Figure 4A). When AtGALT31A-GFP was expressed alone, it was immunoprecipitated as a band of ca. 70 kDa using Western blot analysis with the same antibody (Figure 4A, lane 2). The corresponding band was also detected in the immunoprecipitated material using anti-HA resin from the co-expression sample of both proteins (Figure 4A, lane 5). This indicates co-purification of AtGALT31A with AtGALT29A using a tag on AtGALT29A, thus the complex formation indicated by the FRET analysis was also confirmed by co-immunoprecipitation (Figure 3). The band around 50 kDa detected in lanes 3-5 is the heavy chain of the HA antibody used for immunoprecipitation, which was somehow detected by the secondary antibody in the Western blot analysis.

We attempted to evaluate the purity of the protein complex(es) by eluting the immobilized complex(es) from the anti-HA agarose slurry using low pH buffer as recommended by the manufacturer; however, the majority of the proteins were not eluted to the buffer in an amount detectable by Western blot analysis (data not shown). When the immunoprecipitated samples collected on anti-HA antibody-agarose were directly subjected to SDS-PAGE and analyzed by the Western blot, we could detect the recombinant proteins (Figure 4).

Using the immunoprecipitated enzyme complex, we investigated GalT activity in the biosynthesis of type II AG using UDP-[^14^C]-Gal as donor-substrate and SP_32_-GFP as acceptor, which is microsomes prepared from *N. benthamiana* after expression of a consensus motifs for AG glycosylation, repetitive Ser-Pro [28]. This material contains various AG oligosaccharides similarly as detected in GAGP_8_ (see method). The protein complex containing AtGALT29A and AtGALT31A exhibited a higher level of [^14^C]-Gal incorporation to the SP_32_-GFP acceptor compared to AtGALT29A alone (Figure 4B). While such an increase was not observed for the combination of AtGALT31A/AtGLCAT14A and AtGALT29A/AtGLCAT14A (lane 6 and 7 in Figure 4B), indicating the increase of enzyme activity is specific by the combination between AtGALT29A and AtGALT31A.

Moreover, the enzyme complex showed higher levels of [^14^C]-Gal incorporation also towards β-1,3-galactan acceptor by the enzyme complex compared to AtGALT29A alone (lane 8-10 in Figure 4B). The results indicate an increase of GalT activity towards both SP_32_-GFP and β-1,3-galactan AG acceptors by the enzyme complex containing AtGALT31A and AtGALT29A when compared to a single enzyme.

### The enzyme complex containing AtGALT31A and AtGALT29A exhibited increased β-1,6-GalT activity adding Gal residues at *O*6 positions of β-1,6-galactan and to β-1,3-galactan

**Figure 5:**
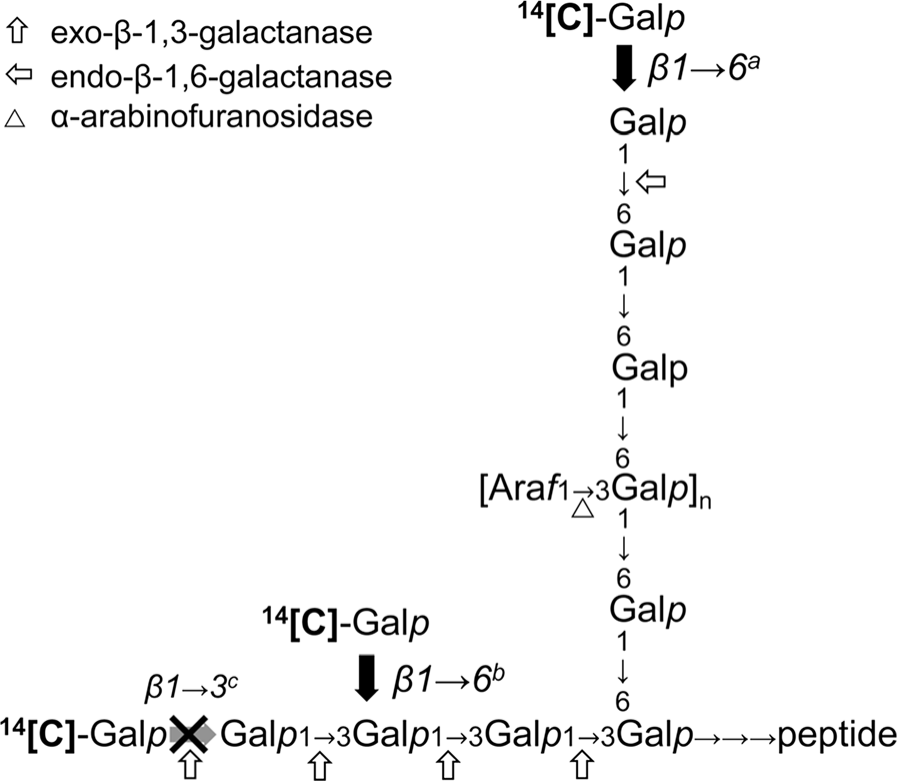
**Simplified model structure of arabinogalactan and reaction sites of enzymes.** The cleavage sites of the hydrolases (exo-β-1,3-galactanase, endo-β-1,6-galactanase, α-arabinofuranosidase) used in this paper are indicated. Recombinant AtGALT29A produced Gal incorporated products susceptible to the treatment of endo-β-1,6- and exo-β-1,3-galactanases (Figure 6), therefore three possible sites (β1 → 6^a, b^ and β1 → 3^c^) are conceivable as the candidate sites of reaction. Towards β-1,3-galactan acceptor, both β1 → 6^b^ and β1 → 3^c^ galactosyltransferase activities are possible, but the main compound released by the exo-β-1,3-galactanase treatment was galactobiose, and not galactose (inset TLC in Figure 6C, D), indicating a β1 → 6^b^ activity rather than β1 → 3^c^ activity. Together with the β1 → 6^a^ activity indicated by the endo-β-1,6-galactanase treatment, it is concluded that, AtGALT29A possesses β-1,6-galactosyltransferase activities both on β-1,3- and β-1,6-galactan (β1 → 6^a, b^ activities).

**Figure 6:**
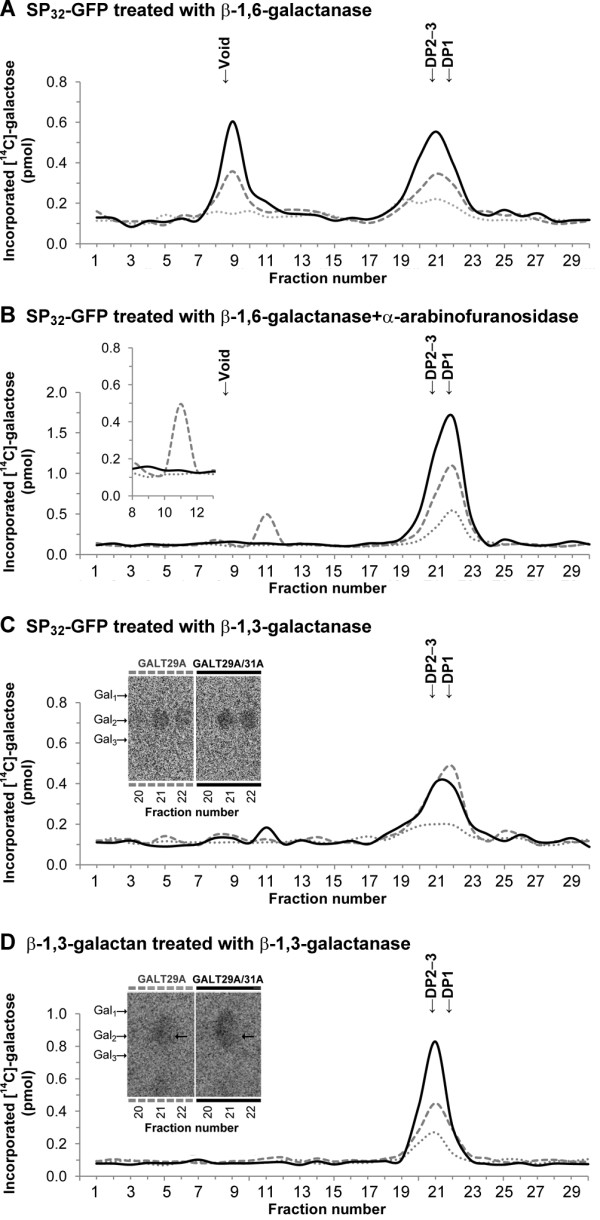
**Analysis of the sites of Gal incorporation in the products produced by AtGALT29A alone or the AtGALT29A/AtGALT31A complex.** The [^14^C]-Gal incorporated products onto SP_32_-GFP **(A, B, C)** or onto β-1,3-galactan **(D)** from P19 [∙∙∙], HA-AtGALT29A [---], or co-immunoprecipitated HA-AtGALT29A/AtGALT31A-GFP complex [▬] were treated with **A**: endo-β-1,6-galactanase, **B**: endo-β-1,6-galactanase + α-arabinofuranosidase, **C**: exo-β-1,3-galactanase, or **D**: exo-β-1,3-galactanase, and separated by size exclusion chromatography using Superdex Peptide HR 10/30. The [^14^C]-Gal present in each fraction was evaluated by scintillation counting. Endo-β-1,6-galactanase, α-arabinofuranosidase, and exo-β-1,3-galactanase used in this study cleave β-1,6-linked unsubstituted galactotriose, terminal α-linked arabinofuranose, and β-1,3-linked galactooligosaccharides regardless the presence or absence of substitutions, respectively. Release of small [^14^C]-oligosaccharides by endo-β-1,6-galactanase indicates the [^14^C]-Gal incorporation to a part of β-1,6-galactotriose, while exo-β-1,3-galactanase releases [^14^C]-Gal monomer from β-1,3-linked galactan and [^14^C]-oligosaccharide (s) from side chains attached to β-1,3-linked galactan. From the [^14^C]-products made onto SP_32_-GFP and β-1,3-galactan, exo-β-1,3-galactanase released mainly [^14^C]-galactobiose analyzed by TLC (inset **C** and **D**), indicating the incorporation of single [^14^C]-Gal to β-1,3-linked Gal at *O*6 in the [^14^C]-products. From any treatments **(A-D)**, higher amount of small [^14^C]-oligosaccharides are released from the [^14^C]-products made by AtGALT29A/AtGALT31A complex compared to that from a single enzyme. The results indicate that AtGALT29A possesses β-1,6-GalT activities elongating β-1,6-galactan and forming 6-Gal branches on β-1,3-galatan, and the β-1,6-GalT activities are increased when AtGALT29A is in a protein complex with AtGALT31A.

The SP_32_-GFP and β-1,3-galactan used in Figure 4 are composed of heterogeneous oligosaccharides: SP_32_-GFP prepared from microsomes consists of various components with different molecular size (ca. 40 kDa, 75-100 kDa, larger than 150 kDa) and contains β-1,6-galactan side chains of a degree of polymerization (DP) from 1 to at least 8, as well as unsubstituted β-1,3-linked galactan [18]. In contrast, β-1,3-galactan acceptor is approximately 25 kDa and consists mostly of unsubstituted β-1,3-galactan (DP 154) with trace amount of β-1,6-linked Gal [29]. Galactose could be incorporated in the AGP molecule at different sites: at *O*3 of β-1,3-galactan (β-1,3^c^-GalT elongating β-1,3-galactan main chain), at *O*6 of β-1,3-galactan (β-1,6^b^-GalT making 6-branches on β-1,3-galactan) and/or *O*6 of β-1,6-galactan (β-1,6^a^-GalT elongating β-1,6-galactan side chains; Figure 5). We investigated the site of the [^14^C]-Gal incorporation catalyzed by the recombinant proteins among the above mentioned possibilities by treating the [^14^C]-Gal incorporated products made onto SP_32_-GFP and β-1,3-galactan with structure-specific hydrolases and subsequent size exclusion chromatography (Figure 6). The endo-β-1,6-galactanase and exo-β-1,3-galactanase used in this study specifically cleave unsubstituted β-1,6-linked galactooligosaccharides of DP3 or longer [30] and β-1,3-linked galactose regardless the presence of substitutions [31], respectively.

From the product made onto SP_32_-GFP, the treatment with endo-β-1,6-galactanase alone released large amounts of material eluting in the void volume, as well as small oligosaccharides with a peak at fraction 21, corresponding to DP2-3, from both the AtGALT31A/AtGALT29A complex and AtGALT29A alone (Figure 6A). The material in the void volume in Figure 6A was almost completely digested by co-treatment with endo-β-1,6-galactanase and α-arabinofuranosidase (Figure 6B), indicating a part of [^14^C]-Gal incorporation occurred at the β-1,6-linked galactans substituted with Ara, and that Ara substitution sterically hindered the action of endo-β-1,6-galactanase [30]. The results indicate that both the enzyme complex and AtGALT29A alone incorporated [^14^C]-Gal to both Ara-substituted and non-substituted β-1,6-galactans, and the level of total Gal incorporation to both types of acceptors was much higher with AtGALT29A in a complex with AtGALT31A. AtGALT31A was previously characterized using radish AGP as acceptor for the incorporation of [^14^C]-Gal and the product was digested by endo-β-1,6-galactanase [17]. We tested the GalT activity of AtGALT31A using SP_32_-GFP acceptor used in this study and showed that the level of activity of AtGALT31A alone was lower than the level observed for the AtGALT29A alone (Additional file 3: Figure S3). Hence, the overall results indicate a cooperative action of GalT activity in elongating β-1,6-galactan of type II AG by forming an enzyme complex containing AtGALT29A and AtGALT31A.

Treatment with exo-β-1,3-galactanase to the products made onto SP_32_-GFP released small oligosaccharides eluting at fraction 22 and 21 as a peak by AtGALT29A alone and by AtGALT29A in a complex with AtGALT31A, respectively (Figure 6C). Both fractions contained galactobiose as the major component analyzed by TLC, but the amount was much higher from the product made by the AtGALT29A/AtGALT31A complex (Figure 6C, inset). Since exo-β-1,3-galactanase cleaves β-1,3-linked Gal, the detected galactobiose is likely β-1,6-linked single Gal substituted onto β-1,3-linked Gal. Thus, the results indicate that both AtGALT29A alone and the AtGALT29A/AtGALT31A complex likely transfer Gal to *O*6 position of β-1,3-linked galactan, and that the amounts of [^14^C]-Gal transfer was higher by the AtGALT29A/AtGALT31A complex.

The GalT activity towards β-1,3-linked Gal was further investigated using β-1,3-galactan as acceptor (Figure 6D, [29]). When the products made on β-1,3-galactan were treated with exo-β-1,3-galactanase [31], the main peak appeared at fraction 21 (Figure 6D) and much more [^14^C]-Gal containing compound was released from the product made by AtGALT29A/AtGALT31A complex compared to AtGALT29A alone. The major component released was galactobiose as indicated by TLC (Figure 6D, inset) and the higher level of [^14^C]-galactobiose was detected from the product produced by the AtGALT29A/AtGALT31A complex, which is consistent with the result obtained from SP_32_-GFP analysis (Figure 6C). Therefore, we confirmed that the GalT activity onto β-1,3-galactan is mainly a branch forming activity (β-1,6-GalT) and this activity is significantly increased by the AtGALT29A/AtGALT31A complex compared to AtGALT29A alone.

Taken together, analysis of the enzymatic activities indicates that AtGALT29A alone has a β-1,6-GalT activity for elongating β-1,6-galactan and forming 6-Gal branches on β-1,3-galactan, and these activities are significantly increased when AtGALT29A is in a complex with AtGALT31A.

### *N. benthamiana* microsomes showed increased galactose incorporation to endogenous type II AGs after co-expression of AtGALT31A and AtGALT29A

**Figure 7:**
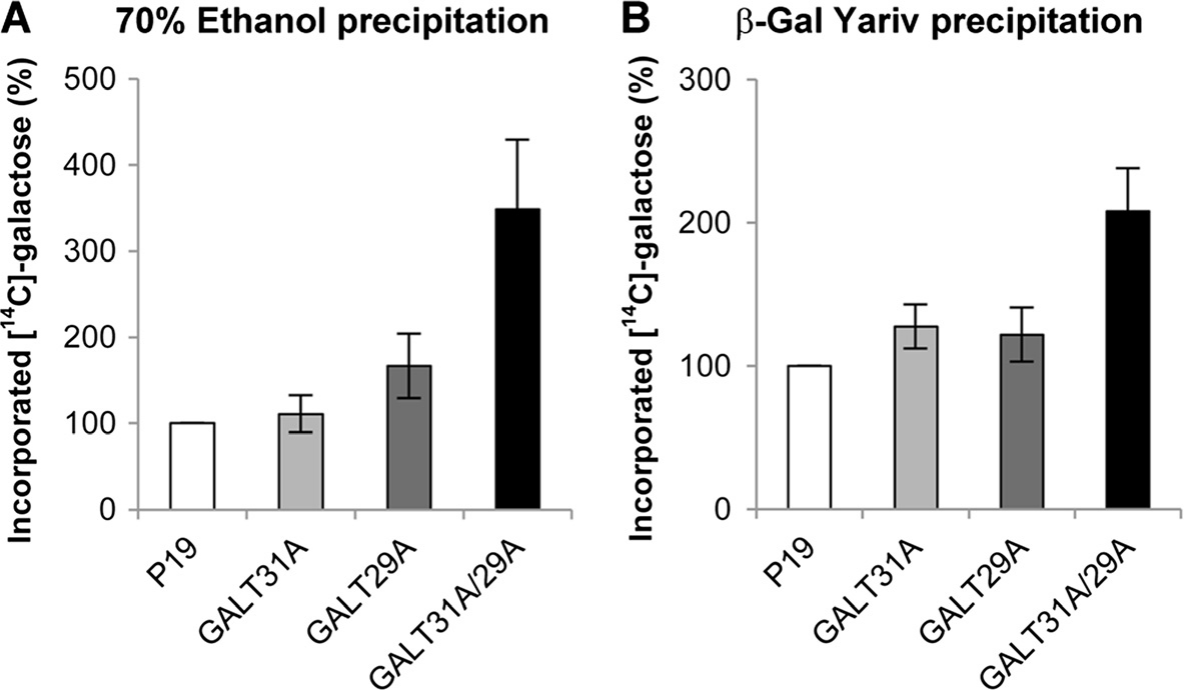
**Galactosyltransferase activity in intact microsomes isolated from *N. benthamiana *after co-expression of *HA*-*AtGALT29A *and *AtGALT31A-GFP*.** Microsomes were incubated with exogenously added UDP-[^14^C]-Gal and the [^14^C]-Gal incorporation to luminal endogenous materials were analyzed by precipitation either by **A**: 70% ethanol or **B**: β-Gal Yariv reagent. Error bars showed standard deviations from n = 4.

Since *in vitro* analysis suggested an increase of the enzyme activity when AtGALT29A is in a complex with AtGALT31A, we also studied possible *in vivo* effects of co-expression of *AtGALT31A* and *AtGALT29A* for AGP glycosylation activity in *N. benthamiana*. We isolated microsomes after co-expression of both proteins and tested incorporation of exogenously added UDP-[^14^C]-Gal to endogenous type II AG, mediated via endogenous UDP-Gal transporter(s) and GalTs present in the lumenal side of vesicles [32]. The synthesis of type II AG products was investigated by [^14^C]-Gal incorporated polysaccharide materials precipitated by 70% ethanol (Figure 7A), or by type II AG precipitated by the β-Gal-Yariv reagent (Figure 7B). In both cases, the results indicated a higher level of Gal incorporation to the polysaccharide materials and β-Gal-Yariv precipitates in the microsomes after co-expression of *AtGALT31A* and *AtGALT29A* compared to expression of each. Thus, the co-expression of *AtGALT31A* and *AtGALT29A* in *N. benthamiana* increases the Gal incorporation activity to endogenous type II AG materials in isolated microsomes.

## Discussion

### Identification of glycosyltransferases involved in the biosynthesis of type II arabinogalactan

In this paper we have shown that the protein encoded by Arabidopsis *At1g08280* gene is a β-1,6-GalT that is involved in the glycosylation of type II AG. We hypothesized that the enzyme is a putative GT involved in the biosynthesis of type II AG based on co-expression analysis together with two other GT genes previously identified in the same glycosylation pathway (*AtGALT31A* and *AtGLCAT14A*) [17,18]. This may appear surprising since the GT belongs to the GT29 family and the protein sequence encoded by *At1g08280* contains ‘sialyl motifs’ conserved in sialyltransferases in mammals and fungi [20]. Sialyltransferase activity was previously tested for the protein encoded by *At1g08280* and concluded to be negative [21]. Apparently the sialyl motifs do not work as independent domains, since a chimeric protein constructed with a sequence encoded by Arabidopsis *At3g48820* and the sialyl motifs from human sialyltransferase did not result in sialyltransferase activity [22]. The GT29 proteins from Arabidopsis (3 proteins in *Arabidopsis thaliana*) and rice (5 proteins in *Oryza sativa*) share homologous sequences and all contain putative sialyl motifs; however, only two of the rice proteins demonstrated sialyltransferase-like activity [21], while two Arabidopsis proteins did not [21,22]. Thus, proteins harboring sialyl motifs apparently do not necessarily encode an enzyme with sialyltransferase activity.

It is difficult to predict the biochemical activity of putative GTs by analyzing the primary sequences, but co-expression studies based on genome-wide expression data in *A. thaliana* (e.g., GeneCAT) [23] were useful in identifying putative candidate GTs involved in type II AG biosynthesis. We selected *AtGLCAT14A* and *AtGALT29A* based on the co-expression profile with *AtGALT31A* and characterized as biosynthetic enzymes involved in type II AG glycosylation. Co-expression analysis using genes encoding the protein core for type II AG modification as markers has been established [33], which may be a good resource to investigate the rest of the pathway. In order to identify the biochemical activity of the putative GT candidates, we established screening methods to cover broad activities expected to be involved in the biosynthesis of type II AG (Figure 1). We found microsomal materials after expression of SynGMs in *N. benthamiana* quite useful for donor substrate identification as they contain a mixture of various oligosaccharides present in type II AG. Otherwise, structure-defined oligosaccharides are difficult to obtain from commercial sources, and even if available, they are expensive and only useful for a specific GT assay. Using the microsomal materials mentioned above as the acceptor mixture, we screened donor substrates for the recombinant enzyme expressed in *N. benthamiana.* The strategy worked for the characterization of AtGALT31A [17], AtGLCAT14A [18], and AtGALT29A (Figure 1), and is expected to be useful to analyze other unidentified GTs in the type II AG glycosylation pathway.

In this paper, we reported that AtGALT29A possesses β-1,6-GalT activities for elongating β-1,6-galactan and forming 6-Gal branches on β-1,3-galactan. Furthermore, AtGALT29A forms enzyme complex together with AtGALT31A, and the complex showed significantly higher level of β-1,6-GalT activities exhibited by AtGALT29A alone.

### Impact of the protein complexes in the glycosylation processes

Based mainly on the studies using yeast and mammalian enzymes, evidence of protein-protein interactions among GTs has been accumulated, namely, that several GTs can form homomeric complexes with themselves and/or interact with other GTs or non-GT proteins via heteromeric complexes (for review see [34]). The complex formation is considered to serve various biological significances, e.g., activate/stabilize the catalytic activity, alternate the substrate specificity, allow proper targeting, and control the localization in ER/Golgi apparatus. In addition, the clusters of GTs are considered to be an assembly line for the efficient and accurate production of certain glycoforms by substrate channeling (for reviews see [35,36]). In plants, evidence for protein-protein interactions between GTs in the secretory pathway are emerging for the biosynthesis of pectin (GAUT1 and GAUT7 [37], (ARAD1 and ARAD2 [38]), xyloglucan (CSLC4, XXT1/XXT2, and XXT5) [39,40], glucuronoarabinoxylan (IRX10 and IRX14) [41], and protein *N*-glycosylation (GMI, GnTI, GMII and XylT) [42]. A putative interaction is also implicated from the cooperative activity and/or co-expression profile in the biosynthesis of galactomannan (ManS and GMGT) [43], xylan (IRX9 and IRX14) [44,45] and mannan (CSLD2 and CSLD3) [46]. The interaction of GAUT1 to GAUT7 has been demonstrated to be important to target catalytic domain of GAUT1 to the Golgi [37], but besides this study, little is known for the significance of forming protein complex(es) among GTs in plants.

In this paper, we evidently demonstrate the presence of homodimeric interactions between for both, AtGALT29A and AtGALT31A by FRET analysis, and do this also for heterodimeric ones between AtGALT31A and AtGALT29A, when these proteins were ectopically expressed in *N. benthamiana* leaves (Figure 3). Moreover, AtGALT31A-YFP could biochemically be co-immunoprecipitated using HA antibody against HA epitope tagged N-terminally to AtGALT29A (Figure 4), and the protein complex(es) containing AtGALT31A-YFP and HA-AtGALT29A exhibited an increased level of β-1,6-GalT activities compared to HA-AtGALT29A alone (Figure 6). Therefore, the complex formation may have a regulatory role in the β-1,6-galactan biosynthesis in type II AG. Accordingly, the present study offers one of the few examples showing a biological significance in the molecular interaction between GTs in plants. It is conceivable that the regulation of biosynthesis via formation of protein complexes among biosynthetic enzymes is faster than transcriptional regulation, and that this mode allows determining subtle changes of cell-surface type II AG structures during cell differentiation in plants. How common such a system for other GTs involved in the biosynthesis of type II AG remains to be elucidated.

According to different levels of FRET efficiencies among different combination of AtGLAT29A and AtGLAT31A, tagged with mCER3 and YFP and reciprocally, respectively, we suggest that AtGALT31A is less capable of dimerization, while AtGALT29A forms dimers more effectively than AtGALT31A. Furthermore, formation of heterodimers between AtGALT31A and AtGALT29A seems to be more dominant than that of homodimers when both AtGALT31A and AtGALT29A are available. With increasing probability we suggest occurrence of dimerization in following sequence: AtGALT31A monomer, AtGALT31A homodimer, AtGALT29A homodimer, and finally AtGALT31A/AtGALT29A heterodimer.

Since the FRET efficiencies might be influenced by the protein stoichiometry in the Golgi stacks, we tried to quantify the proteins expressed ectopically in *N. benthamiana*, but failed because of the low level of protein expression. We could not detect the expressed proteins in *N. benthamiana* microsomes analyzed by SDS-PAGE followed by Western blot. Neither did Native-PAGE lead to detectable amounts in Western blots (data not shown). Therefore we could neither normalize the FRET efficiencies based on the protein concentration nor detect protein complexes under the experimental condition used. However, acceptor photobleaching, which is the method used for calculating the FRET efficiencies in the present study, is quite robust against differences in expression of the two FRET partners, when compared to sensitized emission [26]. Eventually, immunoprecipitation of the proteins in microsomes from *N. benthamiana* allowed us to detect the recombinant proteins by Western blot analysis (Figure 4).

## Conclusions

The *AtGALT29A* (*At1g08280*) from *Arabidopsis thaliana* encodes a β-1,6-GalT involved in the biosynthesis of type II AG by heterologous expression of the protein in *N. benthamiana* and the biochemical enzyme assay. When expressed simultaneously, AtGALT29A interacted with AtGALT31A, and the enzyme complex exhibited substantially increased level of β-1,6-GalT activities compared to AtGALT29A alone. The complex formation could be an important regulatory mechanism for producing β-1,6-galactan side chains of type II AG during plant development.

## Methods

### Materials

Full-length *At1g08280* cDNA with and without stop codon cloned into the Gateway vector, pDONR221 and pDONR223, respectively, were the kind gifts of Dr. Masood Z. Hadi (Joint BioEnergy Institute, Lawrence Berkeley National Laboratory). Plasmids encoding synthetic glycomodule peptides of AGP in a pBI121 vector (SynGMs: GAGP_8_ and SP_32_) [24,28] were the kind gifts of Dr. Marcia Kieliszewski (Ohio University). Preparation of endo-β-1,6-galactanase from *Streptomyces avermitilis* (Sa1,6Gal5A) [30] and exo-β-1,3-galactanase from *Phanerochaete chrysosporium* (Pc1,3Gal43A) [31] followed the procedure described in the publications. Radiochemicals were from PerkinElmer (Boston, MA). UDP-Xyl was from CarboSource (Complex Carbohydrate Resource Center), and other nucleoside diphosphate (NDP) sugars were from Calbiochem-Novabiochem. Other chemicals were from Sigma-Aldrich unless otherwise specified.

### DNA constructions

For enzyme assays, full-length *At1g08280* cDNA containing a stop codon cloned in pDONR221 was moved into pEarleyGate 201 vector [47] to create a hemagglutinin (HA) fusion tag at the N-terminus using LR clonase II (Invitrogen, Life Technologies, Carlsbad, CA). Generation of a C-terminal GFP fusion construct for AtGALT31A (At1g32930) in the pGWB6 vector is described in [17]. For microscope analyses, full-length cDNA sequences without a stop codon cloned in pDONR223 were moved into a modified pEarleyGate vector containing monomeric CFP (pEarleyGate mCer3; vector construction as described in [18]) and pEarleyGate 101 [48] to generate C-terminal mCer3-HA and YFP-HA fusions, respectively. Expression constructs were transformed into *Agrobacterium tumefaciens* strain C58C1 pGV3850 for expression in *N. benthamiana*. Full-length *At5g39990* (*AtGLCAT14A*) [18] cDNA containing a stop codon cloned in pDONR221 was moved into pEarleyGate 201 vector as described above.

### Expression of recombinant proteins in *N. benthamiana*

Infiltration of *N. benthamiana* leaves with *Agrobacterium* strain(s) harboring the appropriate GT(s) was always performed as co-infiltration with the strain harboring the *p19* construct as described in [17]. The p19 protein derived from tomato bushy stunt virus works as a suppressor of gene silencing in the Agrobacterium-mediated transient gene expression system [49]. For enzyme assays, *N. benthamiana* leaves were co-infiltrated with *Agrobacterium* strains at a final cell density of OD_600_ = 0.4. For the negative control, only the *Agrobacterium* strain harboring the *p19* construct at a cell density of OD_600_ = 0.2 was infiltrated. The infiltrated plants were grown in a greenhouse (28°C/day, 25°C/night with a 16 h photoperiod) and harvested at 4 days post-infiltration. For microscope analyses, *N. benthamiana* leaves were co-infiltrated using the procedure described in [38] with *Agrobacterium* strains at a final cell density of OD_600_ = 0.5. The infiltrated plants were grown in a growth chamber (25°C with 16 h photoperiod, 70% humidity) for 50 hours prior to analysis.

### Purification of recombinant enzymes and enzyme complexes

Preparation of the microsome after expression of the recombinant enzymes followed the procedure described in [17]. The total protein concentration of microsome solutions was adjusted to 5 μg/μL and treated with *n*–dodecyl β–maltoside (final concentration of 5 mM). To affinity purify the GFP fusion proteins, detergent-treated microsomal membranes (1 mg total protein) was incubated with 0.8 μg anti-GFP from mouse IgG_1_κ (Roche Diagnostics, Indianapolis, IN) at 4°C for 2-3 h with rotation followed by addition of 20 μL of protein G agarose slurry (contain 50% resin, pre-equilibrated in PBS) and additional incubation overnight at 4°C. For HA affinity purification, detergent-treated microsomal membranes (1 mg total protein) was incubated with 20 μL of monoclonal anti-HA agarose slurry (containing 50% resin) equilibrated in PBS with rotation for overnight at 4°C. In both treatments, the enzyme-immobilized resin was collected by centrifugation at 500 × g, 30 sec., at 4°C followed by three washing steps in PBS. The enzyme-immobilized resin was suspended in an equal volume of 50 mM HEPES, pH 7.0 with 10% glycerol [17] and used immediately for enzyme assay.

### Preparation of AG acceptors (GAGP_8_-GFP, SP_32_-GFP, and β-1,3-galactan)

Preparation of the microsome after expression of AG glycopeptides (SynGMs; GAGP_8_-GFP and SP_32_-GFP), is described in [18]. The polysaccharide analysis using carbohydrate gel electrophoresis (PACE) after digestion with the specific exo-β-1,3-galactanase indicated very similar compositions derived from type II AG for the SP_32_-GFP material and GAGP_8_-GFP used previously [17], indicating the presence of β-1,6-galactooligosaccharides with DP 1 to 8, which are partially decorated with Ara, and the presence of unsubstituted main chain β-1,3-galactan for both types of acceptors. β-1,3-Galactan was prepared by three times Smith degradation of Gum arabic [29], which contains mainly β-1,3-linked Gal and a trace amount of β-1,6-linked Gal. Average molecular weight is around 25 kDa, which corresponds to DP of ca. 154.

### Enzyme assay

The enzyme assays substantially followed the methods described in [17]. For identification of the donor-substrate, the reaction was performed in the presence of combined or individual NDP-sugars as described in [17,18]. The reaction was performed in the presence of 0.1 mM NDP-sugar (containing 277.5 Bq of NDP-[^14^C-]-sugar), 28 mM HEPES, 10 mM MnCl_2_, pH 7.0, and 5 μL of enzyme-immobilized resin and 5 μL of GAGP_8_–GFP (5 μg/μL) as the acceptor. The reaction was performed at 22°C for 16 h and the products were precipitated in the presence of 0.25 μL of 10 mg/ml horseradish peroxidase and 0.28 μL of 0.3% H_2_O_2_[47]. The presence of [^14^C]-sugars in the pellet was determined by scintillation counting after washing several times with water.

In case the product was further analyzed by hydrolases, the reaction was performed in the presence of higher amount of UDP-[^14^C]-Gal, using 5 μL of enzyme-immobilized resin with 5 μL of SP_32_-GFP (5 μg/μL) or 4 μL of β-1,3-galactan (1 mM) in the presence of 1480 Bq UDP-[^14^C]-Gal, 28 mM HEPES, 10 mM MnCl_2_, pH 7.0 in a total assay volume of 25 μL.

The enzyme assay using intact microsomes followed the method described in [34] in a total assay volume of 25 μL. After 1 h incubation at 25°C, 250 μL of water was added and the mixture was sonicated for 10 sec to burst the microsomal vesicles. [^14^C]-incorporated products were precipitated either by 70% (v/v) ethanol at -20°C for 30 min or β-galactosyl Yariv reagent (10 μL of 10 mg/mL β-Gal-Yariv in the presence of 150 mM NaCl, Biosupplies) at 4°C overnight. The precipitated materials were collected by centrifugation at 10,000 × g, 12°C for 15 min followed by washing three times with 70% ethanol or 150 mM NaCl prior to scintillation counting.

### Product analysis

The products made onto SP_32_-GFP acceptor were collected by incubating with 1 μL anti-GFP monoclonal antibody (Roche) for overnight at 4°C. An additional 10 μL of protein G-agarose slurry (containing 50% resin) in PBS was added and incubated at 22°C for 1.5 h with rotation. Immunoprecipitated material was collected by centrifugation at 200 × g for 30 sec at 4°C followed by washing three times with 150 mM NaCl. The product made onto β-1,3-galactan was precipitated in 70% ethanol and washed three times with 70% ethanol. Treatments with 0.0022 U endo-β-1,6-galactanase and 0.02 U exo-β-1,3-galactanase in 80 mM McIlvaine buffer at pH 5.5 and 4.5, respectively, are described in [17]. Co-treatment of the product with α-arabinofuranosidase (0.08 U, Megazyme) was performed in 80 mM McIlvaine buffer at pH 5.5, together with 0.0022 U endo-β-1,6-galactanase. The hydrolyzed products were applied to a Superdex Peptide HR 10/30 column (GE Healthcare) and eluted by 50 mM ammonium formate (flow rate: 0.4 mL/min, 2 min/fraction). The [^14^C]-sugars in the fractions were analyzed by scintillation counting.

Thin layer chromatography (TLC) was performed by the samples developed with acetonitrile/water (80:20, v/v) onto the TLC plate (Silica gel 60 F_254_; Merck, Darmstadt, Germany). Carbohydrate standards were visualized by H_2_SO_4_/ethanol (10:90, v/v) followed by charring at 120°C and the [^14^C]-Gal was detected using a Phosphor-Imager (Molecular Dynamics Storm 860; GE Healthcare).

### Protein analyses

Determination of the protein concentration, SDS–PAGE and western blotting are described in [18]. Native-PAGE was performed by NativePage Bis-Tris Gel System according to the manufacture (Invitrogen, Life Technologies, Carlsbad, CA).

### Subcellular localization and acceptor photobleaching FRET

After infiltration with *Agrobacterium* harboring appropriate constructs, epidermal cell layers of *N. benthamiana* were analyzed by the method described in [26,27]. The following corrections were used: background subtraction, correction for donor photobleaching during the acquisition cycle (in the range of 1-3%), correction for acceptor cross talk into the donor channel (1-6%), correction for acceptor photoproduct formed upon bleaching (0.5-5%), and correction for the incomplete photobleaching of the acceptor (in the range of 10-40% unbleached fraction). Regions of interest (ROIs) representing Golgi vesicles were segmented as described in [26], and rejected from further analysis if (1) their size was below 4 square-pixels, (2) circularity below 0.3, (3) the percentage of pixels above background in the ROI changed by more than 30% in the post-bleach image, (4) over 30% of their pixels showed out-of-range FRET efficiency, and (5) their averaged FRET efficiency was below -0.05. The pixel-by-pixel distribution of FRET efficiency for each protein combination was generated from pooling all valid ROIs.

## Abbreviations

AGP: Arabinogalactan protein; Ara: α-L-arabinofuranoside; AtGALT29A: *Arabidopsis thaliana* β-galactosyltransferase 1 from family GT29 (At1g08280); AtGALT31A: *A. thaliana* β-galactosyltransferase 1 from family GT31 (At1g32930); AtGlcAT14A: *A. thaliana* β-glucuronosyltransferase 1 from family GT14 (At5g39990); Fuc: Fucose; GAGP8: synthetic glycomodule gene harbouring 8 repetitive 19-residue consensus motif of gum Arabic glycoprotein; Gal: Galactose; GalT: Galactosyltransferase; Glc: Glucose; GlcNAc: *N*-acetyl-D-glucosamine; GlcA: Glucuronic acid; GT: Glycosyltransferase; HA: Hemagglutinin; Man: Mannose; mCer3: Monomeric Cerulean3; NDP: Nucleoside diphosphate; PACE: Polysaccharide analysis using carbohydrate gel electrophoresis; Rha: Rhamnose; ROI: Region of interest; type II AG: arabino-β-1,3-(β-1,6)-galactan; SD: Standard deviation; SP32: Synthetic glycomodule gene harbouring 32 repeats of the Ser-Pro motif; STtmd-YFP: Sialyltransferase short cytoplasmic tail and single transmembrane domain fused to YFP; SynGM: Synthetic glycopeptide/glycomodule; Xyl: Xylose.

## Competing interests

The authors declare that they have no competing interests.

## Authors’ contributions

AD and CPP contributed substantially to design the experiments, to perform the experiments and drafted the manuscript. In particular, AD contributed to the biochemical enzyme assays, purification of the protein complex and its analysis. CPP contributed to the study of subcellular localization and FRET based protein-protein interaction of glycosyltransferases. GV supervised experimental design and data analysis of the FRET acceptor photobleaching study. SK prepared oligosaccharides and specific hydrolases used for the biochemical enzyme assays. AS supervised the use of confocal laser scanning microscopy and guided the FRET analysis. NG conceived and coordinated the project and wrote the manuscript. All authors read and approved the final manuscript.

## Supplementary Material

Additional file 1: Figure S1 Co-expression analysis of *AtGALT29A*, *AtGALT31A* and *AtGlcAT14A*. Click here for file

Additional file 2: Figure S2 Localization and FRET analysis for AtGALT29A and AtGLCAT14A. Click here for file

Additional file 3: Figure S3 Analysis of the products made onto SP_32_-GFP by P19 only control or AtGALT31A. Click here for file
