# Supplementary material for: Galactosyltransferases from Arabidopsis thaliana in the biosynthesis of type II arabinogalactan: molecular interaction enhances enzyme activity
Source: BMC Plant Biol. 2014 Apr 3;14:90. doi: 10.1186/1471-2229-14-90 (PMC4234293; doi:10.1186/1471-2229-14-90)
Supplement: Additional file 2: Figure S2 — Localization and FRET analysis for AtGALT29A and AtGLCAT14A. [file 1471-2229-14-90-S2.pdf]

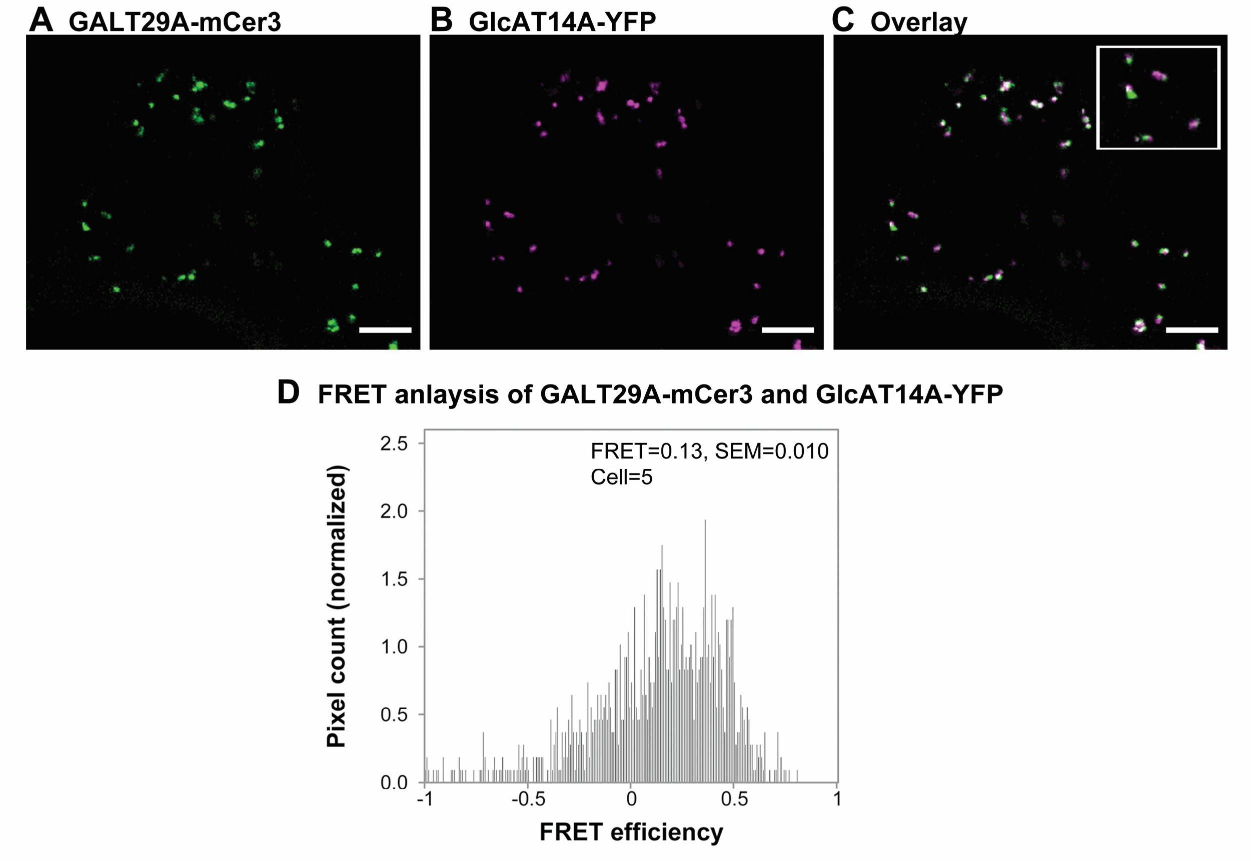

Figure S2. Localization and FRET analysis for AtGALT29A and AtGLCAT14A.

(A-B) Confocal images of AtGALT29A-mCer3 (A) and AtGLCAT14A-YFP (B) co-expressed in *N. benthamiana* leaves. Scale bar = 5 μm.

(C) The overlay image of (A) and (B). AtGALT29A-mCer3 and AtGLCAT14A-YFP often show adjacent localization but only occasionally co-localized (inset, region of not co-localized).

(D) Distribution histogram for pixel by pixel analysis of FRET [2] for AtGALT29A and AtGLCAT14A where they are co-localized. FRET efficiency is 13%, indicating the two proteins can interact when they are co-localized. SEM, standard error of means; cell=number of cells analyzed.
