# Supplementary material for: Galactosyltransferases from Arabidopsis thaliana in the biosynthesis of type II arabinogalactan: molecular interaction enhances enzyme activity
Source: BMC Plant Biol. 2014 Apr 3;14:90. doi: 10.1186/1471-2229-14-90 (PMC4234293; doi:10.1186/1471-2229-14-90)
Supplement: Additional file 1: Figure S1 — Co-expression analysis of AtGALT29A, AtGALT31A and AtGlcAT14A. [file 1471-2229-14-90-S1.pdf]

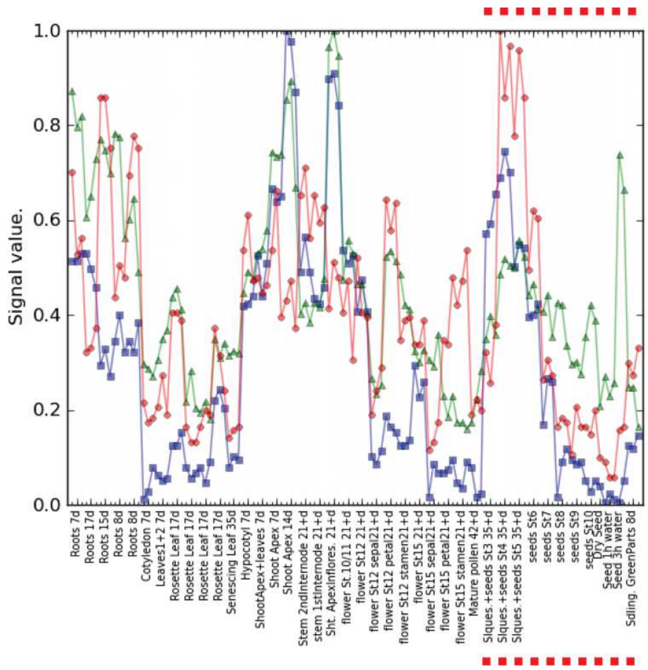

Figure S1. Co-expression analysis of *AtGALT29A*, *AtGALT31A* and *AtGlcAT14A*. Expression profiles for *AtGALT29A* (*At1g08280*, ●), *AtGALT31A* (*At1g32930*, ■) and *AtGlcAT14A* (*At5g39990*, ▲) generated by the GeneCAT (http://genecat.mpg.de) [1]. The expression level of *AtGALT29A* is higher during seed maturation and root development. *AtGALT29A* is highly co-expressed with *AtGALT31A* during seed maturation (indicated by ▪▪▪).
