# Supplementary material for: Galactosyltransferases from Arabidopsis thaliana in the biosynthesis of type II arabinogalactan: molecular interaction enhances enzyme activity
Source: BMC Plant Biol. 2014 Apr 3;14:90. doi: 10.1186/1471-2229-14-90 (PMC4234293; doi:10.1186/1471-2229-14-90)
Supplement: Additional file 3: Figure S3 — Analysis of the products made onto SP32-GFP by P19 only control or AtGALT31A. [file 1471-2229-14-90-S3.pdf]

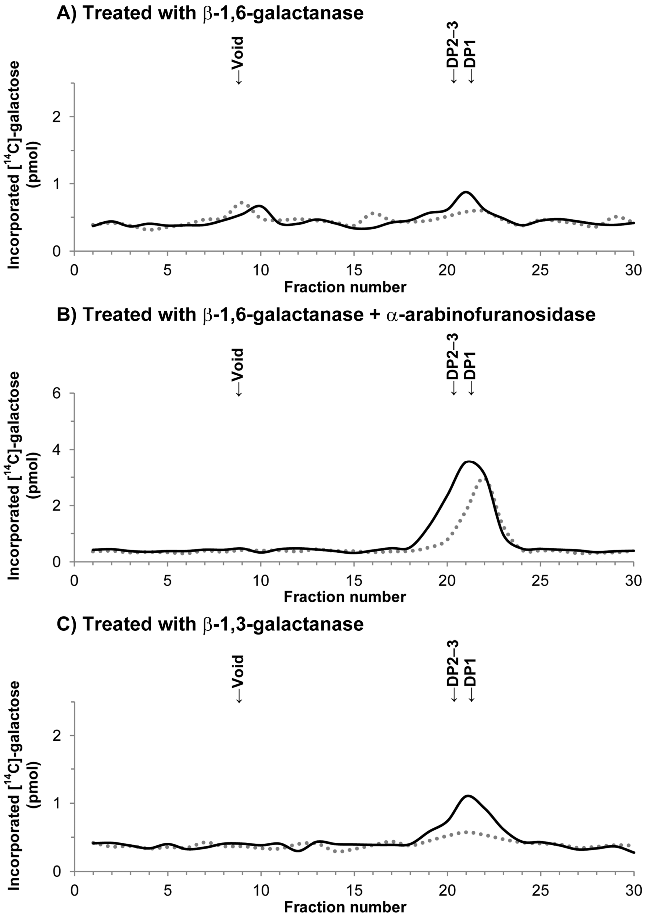

Figure S3. Analysis of the products produced by the P19 only control or AtGALT31A. The same methods used for the analysis presented in Figure 5 were applied for the affinity-purified materials from P19 (···) or AtGALT31A-GFP (▬) using a GFP antibody. The [^{14}C]-Gal incorporated product onto SP_{32}-GFP was immunoprecipitated, treated with endo-β-1,6-galactanase (A), endo-β-1,6-galactanase + α-arabinofuranosidase (B), or exo β-1,3-galactanase (C), and analyzed by size exclusion chromatography using Superdex Peptide HR 10/30. Small amount of oligosaccharides were released by the specific hydrolases, indicating a low level of GalT activity towards β-1,6-Gal and β-1,3-Gal, similar to that found for the AtGALT29A single enzyme (Figure 5A-D).
